# Does in-shoe pressure analysis to assess and modify medical grade footwear improve patient adherence and understanding? A mixed methods study

**DOI:** 10.1186/s13047-022-00600-0

**Published:** 2022-12-24

**Authors:** Clare McDonogh, Vanessa L. Nube, Georgina Frank, Stephen M. Twigg, Stefania Penkala, Samantha Holloway, Robert Snyder

**Affiliations:** 1 Podiatry Department, Royal Prince Alfred Hospital, Sydney Local Health District, Sydney, Australia; 2 Diabetes Centre and Department of Endocrinology, Royal Prince Alfred Hospital, Sydney, Australia; 3 Central Clinical School, Faculty of Medicine and Health and The Charles Perkins Centre, University of Sydney, Sydney, Australia; 4 School of Health Sciences and Translational Health Research Institute, Western Sydney University, Sydney, Australia; 5 College of Biomedical and Life Sciences, Cardiff University School of Medicine, Cardiff, Wales UK; 6 School of Podiatric Medicine, Barry University, Florida, USA

**Keywords:** Diabetes, Foot ulcer, In-shoe pressure analysis, Medical grade footwear, Adherence

## Abstract

**Background:**

Medical grade footwear (MGF) with demonstrated plantar-pressure reducing effect is recommended to reduce the risk of diabetes-related foot ulceration (DFU). Efficacy of MGF relies on high adherence (≥ 80%). In-shoe pressure analysis (IPA) is used to assess and modify MGF, however, there is limited evidence for the impact on patient adherence and understanding of MGF. The primary aim of this study was to determine if self-reported adherence to MGF usage in patients with previous DFU improved following IPA compared to adherence measured prior. The secondary aim was to determine if patient understanding of MGF improved following in-shoe pressure analysis.

**Methods:**

Patients with previous DFU fitted with MGF in the last 12 months were recruited. The first three participants were included in a pilot study to test procedures and questionnaires. MGF was assessed and modified at Week 0 based on findings from IPA using the Pedar system (Novel). Patients completed two questionnaires, one assessing patient adherence to MGF at Week 0 and Week 4, the other assessing patient understanding of MGF before and after IPA at week 0. Patient understanding was measured using a 5-point Likert scale (strongly disagree 1 to strongly agree 5). Patient experience was assessed via a telephone questionnaire administered between Weeks 0–1.

**Results:**

Fifteen participants were recruited, and all completed the study. Adherence of ≥ 80% to MGF usage inside the home was 13.3% (*n* = 2) pre-IPA and 20.0% (*n* = 3) at Week 4. Outside the home, ≥ 80% adherence to MGF was 53.3% (*n* = 8) pre-IPA, and 80.0% (*n* = 12) at Week 4. Change in scores for understanding of MGF were small, however, all participants reported that undergoing the intervention was worthwhile and beneficial.

**Conclusions:**

Self-reported adherence inside the home demonstrated minimal improvement after 4 weeks, however, adherence of ≥ 80% outside the home increased by 27%, with 80% of all participants reporting high adherence at Week 4. Participants rated their learnings from the experience of IPA as beneficial.

**Supplementary Information:**

The online version contains supplementary material available at 10.1186/s13047-022-00600-0.

## Introduction/Background

Diabetes-related foot ulceration (DFU) is a severe and potentially devastating complication of diabetes mellitus with a lifetime incidence in people with diabetes estimated to be between 19%-34% [1]. Concerningly, recurrence rates for DFU are high, reported to be 40% after one year and 65% after three years [1]. A significant factor in the development of DFU is repetitive trauma from plantar pressures that remain undetected due to peripheral neuropathy [2]. A primary strategy in preventing re-ulceration in patients with a history of DFU is the provision of medical grade footwear (MGF) and custom moulded orthoses that accommodate foot deformity and offload high pressure regions [3, 4].

While the provision of MGF in patients with a history of DFU has historically necessitated a trial-and-error approach [5], in-shoe pressure analysis provides the ability to analyse plantar pressures and modify footwear to optimise offloading, with reported mean peak pressure (MPP) reduction ranging from 23%-30.2% [5, 6]. Current national and international guidelines provide a strong recommendation for patients with a history of DFU to be fitted with MGF that demonstrates plantar pressure reducing capacity to reduce the risk of foot ulcer recurrence [3, 4, 7]. International guidelines define plantar pressure reduction as ≥ 30% reduction in peak pressure compared to the current footwear, or MPP < 200 kPa at the site of previous ulceration (when measured with a validated and calibrated system with sensor size of 2cm^2^) [3].

While MGF optimised through in-shoe pressure analysis has the potential to significantly reduce the risk of re-ulceration, the success of MGF is contingent on patient adherence [8]. A combination of both low in-shoe MPP < 200 kPa and > 80% adherence for all steps taken is a significant determinant of DFU recurrence due to unrecognised repetitive trauma [9]. However, it has been identified that adherence to MGF in this population is low, with 29% of participants reporting significance usage (> 80%) during the day [10], and only 12% inside the home [11]. In light of the low adherence to MGF reported in the literature and the increased risk of DFU associated with low adherence, international guidelines have called for further research into strategies that improve patient adherence [3].

Due to peripheral neuropathy, patients with a history of DFU may lack the ability to detect tissue injury that results from inappropriate footwear usage, and may be less motivated to wear their MGF [12]. Factors such as the appearance and perceived comfort of MGF may pose barriers to MGF that need to be overcome through patient understanding of the benefits of MGF [13]. We hypothesise that participation in a single session of in-shoe pressure analysis and footwear modification (with visual demonstration of pressure areas) may educate and motivate patients to increase adherence.

The primary aim of this study was to determine if participating in a single session of in-shoe pressure analysis and knowing the results improves self-reported adherence to wearing MGF at four weeks. Secondary outcomes were to determine if a single session of in-shoe pressure analysis improves patient understanding of the role of MGF and satisfaction with MGF. Finally, this study explored the barriers to MGF adherence and the patient experience of in-shoe pressure analysis.

## Method

The study was conducted using a mixed methods approach, adopting elements of both quantitative and qualitative design. Questionnaires were administered to capture both numerical data related to adherence and understanding, and descriptive data obtained through open-ended questions exploring barriers to adherence and patient experience.

### Participants and study location

Participants were patients referred from the Sydney Local Health District (SLHD) Podiatry and High Risk Foot Services to undergo in-shoe pressure analysis assessment, a relatively new service implemented at the beginning of 2021 as part of the Royal Prince Alfred Hospital (RPAH) Diabetes Centre High Risk Foot Service. Convenience sampling was used for this study, with patients who met the inclusion/exclusion criteria informed of the study during routine consultation and invited to participate. Patients interested in participating were provided with the Participant Information Sheet, which they were given a minimum of 24 h to read.

Inclusion criteria were Type 1 or Type 2 diabetes mellitus, and peripheral neuropathy diagnosed previously on routine neurovascular assessment (10 g monofilament and vibration perception using tuning fork, neurothesiometer or VibraTip™). All participants had a history of DFU on the plantar surface of the foot. Where participants had a history of multiple plantar ulcerations, all sites were included for assessment in this study. Participants were eligible if they owned MGF and custom orthoses or pre-fabricated accommodative orthoses issued within the last 12 months. MGF was defined according to the criteria described in the recent guidelines by Van Netten et al. [7] and included fully customised or pre-fabricated footwear with features that reduce the risk of DFU, such as extra depth or width, or a modified sole (e.g. rigid forefoot rocker). MGF had to contain removable orthoses to facilitate modifications where required. Footwear greater than 12 months of age was excluded as MGF of patients with a history of DFU is routinely replaced at 12 months within SLHD Podiatry due to material fatigue. A minimum period of two weeks wearing prescribed MGF prior to study commencement was included to allow for gradual and safe introduction of MGF and to allow for baseline adherence to be measured.

Patients with active DFU or Charcot neuroarthropathy were excluded from the study, as were patients considered to be at high risk of falls or unable to walk 10 m with or without assistive devices due to inability to safely complete the walking trials required for pressure measurement. Patients with a forefoot amputation or below- or above-knee amputation were excluded for technical reasons due to inability to collect data from one insole only, and to avoid damage to the Pedar insoles by inserting over an in-shoe filler. A large or raised filler used to accommodate a forefoot amputation would require bending of the insole in a way that may damage the equipment. Where patients had a small or shallow filler or a filler located in such a way that the Pedar insole could be safely positioned, they were included in the study.

### Study procedures

Ethical approval for this study was sought and obtained from both Cardiff University School of Medicine Research Ethics Committee (SREC reference SMREC 20/98) and the RPAH Ethics Review Committee (Protocol number X20-0455) in December 2020. All assessments, including questionnaires, were conducted by the investigator (CM [a podiatrist employed by SLHD]). The questionnaires used in this study were original, as no previously validated questionnaires assessing the topics of patient adherence and understanding of MGF were identified. The questionnaires were developed in consultation with the SLHD Research Electronic Data Capture (REDCap) Data Management team to optimise functionality. The questionnaires were then piloted on the first three participants recruited for the study in order to assess acceptability and make necessary modifications for the final study. All questionnaires were read out to participants by the investigator (CM) from the REDCap database hosted by SLHD and developed by Harris et al. [14, 15]. REDCap is a software platform for data collection and management utilised for research studies in SLHD. Questionnaires were delivered in a standardised manner to minimise potential interviewer bias that Meadows [16] noted may occur if there is variability in delivery between participants.

#### Informed consent and Week 0 data collection

Following arrival at the Week 0 appointment, participants were informed of the potential risks of participating in the study. A foot assessment was undertaken to confirm no active foot ulceration or clinical concern for active Charcot neuroarthropathy before informed consent to participate in the study was gained. Following informed consent, baseline data were collected and entered into the REDCap database.

#### Pre-assessment questionnaires

Prior to in-shoe pressure analysis, two questionnaires were administered to ascertain baseline patient adherence (Pre-assessment Questionnaire A – Table 1) and understanding of the role of MGF (Pre-assessment Questionnaire B – Table 1). Pre-assessment Questionnaire A assessed adherence to MGF both inside and outside the home, and participants were also asked open-ended questions regarding potential barriers to wearing MGF and the reasons for wearing MGF. Participants were informed at the beginning of the Questionnaire that we were referring to use of MGF over the last four weeks. Pre-assessment Questionnaire B consisted of five statements related to loss of protective sensation and use of MGF, and participants were provided with a five-point Likert scale to indicate the extent to which they agreed or disagreed with each statement.

**Table 1:** Pre-assessment Questionnaires A and B

**Pre-assessment Questionnaire A**	
What percentage of the time have you worn the footwear at home?	◦ < 20% ◦ 20–49% ◦ 50–79% ◦ 80–99% ◦ 100% ◦ Unsure ◦ Prefer not to answer
What percentage of the time have you worn the footwear outside the home?	*As above*
What have been the barriers to using your prescribed footwear?	*Free text*
What are the reasons why you have worn your prescribed footwear?	*Free text*
**Pre-assessment Questionnaire B**	
*I am going to read you a series of statements and I would like you to tell me whether you strongly disagree, disagree, feel neutral (have no strong opinion), agree, strongly agree, or prefer not to answer* 1. Because I have diabetes and loss of protective sensation, I may not feel high pressure areas under my feet 2. My footwear and orthotics have been designed to prevent another foot ulcer 3. High pressure areas under my feet may lead to an ulcer if I do not wear my prescribed footwear most (> 80%) of the time 4. Regular shoes may not protect my feet as well as my prescribed footwear 5. It is OK (or safe) to wear slippers or other slip-on house shoes when I am at home as these protect my feet enough	◦ Strongly disagree ◦ Disagree ◦ Feel neutral ◦ Agree ◦ Strongly agree ◦ Prefer not to answer
Are you satisfied with your footwear?	◦ Yes ◦ No ◦ Unsure ◦ Prefer not to answer
*(If yes)* Why are you satisfied with your footwear	*Free text*
*(If yes)* Is there anything about your footwear that you are not satisfied with?	*Free text*
*(If no)* Why are you not satisfied with your footwear?	*Free text*
*(If no)* Is there anything about your footwear that you are satisfied with?	*Free text*
*(If unsure)* Why are you unsure?	*Free text*

#### In-shoe pressure analysis and modification of footwear/orthoses

The Pedar® system (Novel, Germany) was used to conduct in-shoe pressure analysis. Once the Pedar equipment was fitted to the patient, insoles were calibrated according to the manufacturer’s instructions [17]. The procedure for pressure analysis was similar to that previously described by Arts et al. [18]. Participants were asked to walk along a 10-m marked walkway three times to obtain a minimum of 12 midgait steps per foot. Arts and Bus [19] demonstrated that 12 steps are required for valid and reliable in-shoe pressure data in this patient cohort. Participants were asked to walk at their normal everyday walking pace and the speed of subsequent trials was allowed to be within 5% of the original speed. Where subsequent trials were over 5% faster or slower, participants were asked to repeat the trial to keep the speed consistent.

**Fig. 1:**
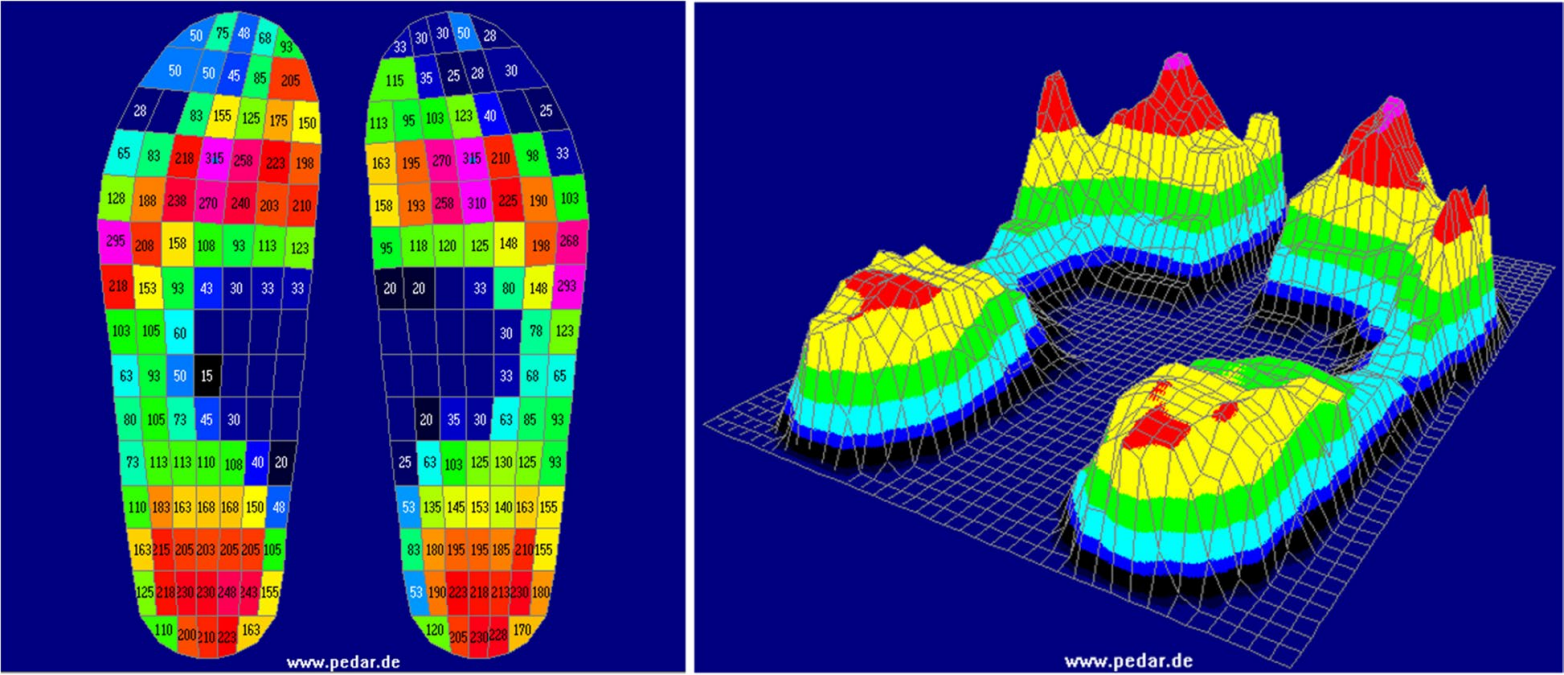
2D and 3D pressure map in the *Pedar-x* program

Data were collected using the *Pedar-x* program where a coloured pressure-map displaying the maximum pressure picture was generated and demonstrated on screen to participants by the investigator (CM) (Fig. 1). The data were then exported to the *Step Analysis* program where unwanted steps were removed (including turning steps). The average maximum pressure picture (consisting of the MPP across all steps) was shown to participants. The site(s) of previous ulceration and any additional areas where MPP > 200 kPa were indicated to participants on the screen and their significance as possible sites of future ulceration was explained in non-technical language.

A maximum of three sites with MPP > 200 kPa per participant were targeted for pressure reduction through orthotic modification, as this was deemed an achievable number to target and was the number of sites selected by Bus et al. [5]. Where more than three sites demonstrated MPP > 200 kPa, the three with the highest MPP were chosen. The site(s) of previous ulceration where MPP > 200 kPa were prioritised, followed by sites demonstrating MPP > 200 kPa that were not sites of previous ulceration. A maximum of three rounds of orthotic modification took place and pressure analysis was conducted after each round to assess for changes in MPP at the region of interest (ROI). Following each round of pressure analysis and modification, the average maximum pressure picture in *Step Analysis* was shown to participants. The impact of the modifications on plantar pressures was explained, and the importance of wearing footwear to benefit from the offloading was reinforced.

Modifications consisted of adding a metatarsal pad or bar, removing material at the ROI and adding cushioning, adding an additional top cover, or adding an extra layer under the orthotic with a deflection to the ulcer site. Modifications were performed by the RPAH orthotists and the investigator (CM). If no sites demonstrated MPP > 200 kPa, a non-customised insole or shoe was used to create a comparison with MGF. This consisted of an extra-depth shoe with no customised insole (where participants brought additional footwear), or a flat 6 mm Plastazote insole (approximate density 0.0399 g/cm^3^) [20] was inserted into the MGF in place of the custom insole to demonstrate the benefit of the customised device.

The highest MPP at a ROI was calculated by identifying the individual sensor with the highest MPP at the given foot region (e.g. first metatarsal head region for a patient with a previous ulcer at this site). It is recognised that previous studies have utilised masking software to create an average MPP at each foot region [5, 6, 18]; however, this capability is not available with the standard level of software, and therefore may not be available to all clinicians using this technology. The process of masking and analysing regions is both technical and time-consuming, and may not be feasible in the time available to clinicians for patient assessment and orthotic modification. Furthermore, the process of masking, while useful for data analysis and research purposes, does not aid in further educating patients through visual demonstration of pressure areas, which was the primary aim of this study. The current study aimed to utilise software and methods that are readily available to clinicians and feasible in the patient setting.

#### Post-assessment questionnaire

Following completion of the pressure analysis session, the Post-assessment Questionnaire was administered. The Post-assessment Questionnaire was identical to the Pre-assessment Questionnaire B (Table 1), and was designed to assess if participants’ responses to the statements indicating their level of understanding of MGF changed or stayed the same following pressure analysis.

#### Week 1 and Week 4 questionnaires

In the week following the Week 0 appointment, a telephone questionnaire (see Results section Table 4) was administered by an investigator other than CM, who conducted the pressure analysis session. This questionnaire was designed to evaluate the patient experience of the pressure analysis session.

Participants attended a follow-up appointment at Week 4. Where possible, this appointment was undertaken face-to-face to facilitate visual assessment of participants’ feet to assess for any lesions. The Week 4 follow-up questionnaire was identical to the Pre-Assessment Questionnaire A and was designed to measure whether there had been any change in self-reported adherence over the four weeks following pressure analysis. Participants who ceased use of MGF during the study were not excluded from follow-up questionnaires unless they requested to be withdrawn from the study. Data from participants who suffered an adverse event or who were not able to continue use of MGF following the intervention were collected to measure outcomes for those who had both positive and negative experiences following pressure analysis.

#### Data analysis

All statistical analyses were carried out using Microsoft Excel (Microsoft Office 2021). To assess the impact of the intervention on patient understanding of the role of MGF, the Likert scale responses for Pre-Assessment Questionnaire B were analysed and compared to the responses for the Post-Assessment Questionnaire. Each Likert scale response was coded with a number to create a score (best possible score 5, worst possible score 1). Paired sample *t-*tests were used to assess the impact of in-shoe pressure analysis on patient understanding by comparing the Pre-assessment Questionnaire B and Post-assessment Questionnaire scores. All tests were applied two-tailed using a significance of *p* < 0.05. The mean score for each statement as well as the overall score before and after the intervention was calculated.

Thematic analysis, as described by Braun and Clarke [21] and Nowell [22], was used to extract keywords or phrases from questionnaire responses, which were then reviewed to generate themes related to non-adherence and adherence to MGF, satisfaction with MGF, and the experience of pressure analysis.

#### Pilot study

The first three participants were included in a four-week pilot study, which employed the same procedures as those utilised for the main study. The purpose was to assess the feasibility and acceptability of the questionnaires, and to determine if sufficient time and procedures were in place to conduct pressure analysis and orthotic modifications.

## Results

Between 1 January and 30 June 2021, 26 patients were identified as eligible and invited to participate. All 26 patients expressed interest in the study, of which 22 agreed to participate and a Week 0 appointment was scheduled. One patient was later deemed ineligible prior to the baseline appointment due to falls risk, while another patient had to cancel for personal reasons unrelated to the study. A further two patients were deemed ineligible upon arrival at the Week 0 appointment due to age of MGF (> 12 months) and active DFU. The number of participants recruited for the pilot study was three and for the final study this was 15. All 18 participants completed the study and follow-up questionnaires; however, eight did not attend the Week 4 appointment in person and the data collection and questionnaire were conducted over the phone. The primary reason for not attending was due to considerations surrounding the COVID-19 pandemic (*n* = 5), followed by forgot appointment (*n* = 1), travel (*n* = 1) and illness (*n* = 1). The pilot study took place between 20 January and 17 March 2021 and the final study between 3 March and 21 July 2021. For data analysis, only the 15 participants in the final study have been included in the results.

### Participant characteristics

Participant characteristics are provided in Additional file 1. Most participants had T2DM (*n* = 14) with a group mean HbA1c% of 7.5% (range 5.3%-11.5%). The majority of participants (*n* = 10) wore modified MGF, with a rigid forefoot rocker the most common modification (*n* = 9). None of the participants had undergone in-shoe pressure analysis previously. Thirty-two previous foot ulcers were reported with the most common location for both feet combined the forefoot (*n* = 15) followed by hallux (*n* = 12). The mean time since the most recent ulcer healed was 28.4 months (range 1–180 months). Most participants (*n* = 13) had not been diagnosed with peripheral arterial disease. All participants had one or more foot deformities affecting one or both feet, with the most common deformities consisting of claw/hammer toes (*n* = 9), hallux limitus/rigidus (*n* = 7), and prominent metatarsal heads (*n* = 7).

### Pressure analysis data

A total of 24 sites where MPP > 200 kPa at baseline were targeted for pressure reduction. Pressure reductions were achieved for all but four sites (see Additional file 2 for the full list of pressure reductions and modifications performed). Following pressure analysis, 8 sites (33%) were successfully offloaded to < 200 kPa, with an average of 2 rounds of orthotic modification required. For two participants there were no sites > 200 kPa and no modifications were required.

### Patient adherence

**Fig. 2:**
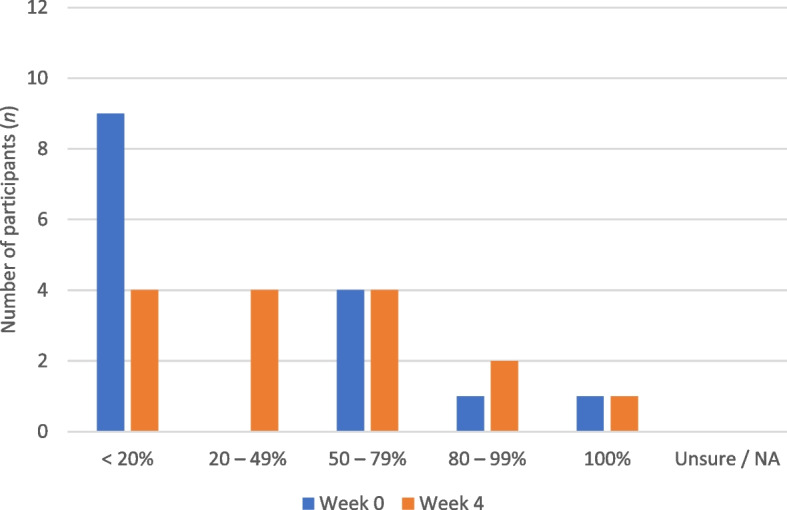
Adherence to MGF inside the home at Week 0 (blue) and Week 4 (orange) (*n* = 15)

The ranges for self-reported adherence inside the home prior to pressure analysis at Week 0 and at Week 4 are presented in Fig. 2. The range of self-reported adherence inside the home increased for seven (46.7%) participants, decreased for one (6.6%), and remained the same for seven (46.7%).

**Fig. 3:**
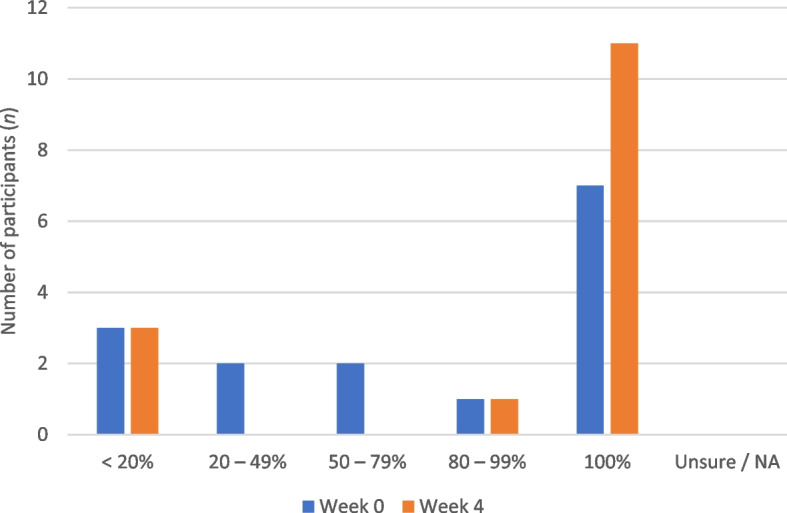
Adherence to MGF outside the home at Week 0 (blue) and Week 4 (orange) (*n* = 15)

The data for adherence outside the home are presented in Fig. 3. The data show that self-reported adherence outside the home increased for five (33.3%) participants and stayed the same for 10 (66.7%), of which seven were already 100% at baseline.

The number of participants who reported ≥ 80% adherence at Week 0 was two (13.3%) for inside the home and eight (53.3%) for outside the home. This increased to three (20.0%) for inside the home and 12 (80.0%) for outside the home at Week 4.

Several themes surrounding non-adherence and adherence to MGF were identified in the responses to the Week 0 Pre-assessment Questionnaire A and Week 4 Questionnaire. The themes for non-adherence are presented in Table 2. The number of times each theme was identified in participant responses at Week 0 and Week 4 is presented. A significant proportion of themes for non-adherence to MGF related specifically to use inside the home.

**Table 2:** Themes for non-adherence and adherence to MGF

	**Number of times theme identified at Week 0**	**Number of times theme identified at Week 4**
**Themes for non-adherence**		
Preference for wearing other shoes at home for some or all of the time (e.g. moving from bed to bathroom or getting up from watching TV)	2^a^	3^a^
Preference for wearing no shoes at home for some or all of the time (including barefoot or in socks)	3^a^	2^a^
Sitting or keeping feet up at home	2^a^	2^a^
Feels safe walking barefoot at home	1^a^	1^a^
Difficulty walking in MGF (e.g. due to unsteadiness or difficulty adjusting)	4	0
Discomfort	4	2
Heaviness	1	1
Difficulty driving	1	0
Difficulty applying and removing MGF	1	1
Appearance	2	0
Concerns with fit	2	2
Cleanliness concerns (indoors)	3^a^	0^a^
Lack of perceived benefit	1	1
Forgets to wear	0	1
Caused skin trauma (e.g. blister or ulcer)	1	3
**Total**	28	19
**Themes for adherence**		
Safety and protection of feet	2	5
Avoiding ulcers and/or amputation	4	2
Following healthcare advice	7	6
Warmth	1	0
Perceived benefit	3	3
Comfort	6	4
Ease of walking	1	0
Accustomed to use	1	0
Appearance	1	0
Preventing blisters and/or callus	2	1
Unsure if able to transfer orthoses to other shoes	1	0
Appreciative of resources	0	2
For work purposes	0	1
Other shoes are not comfortable or cause skin trauma	1	2
**Total**	30	26

^a^Relates specifically to use inside the home

Themes surrounding adherence to MGF are also presented in Table 2. Dominant themes at both Week 0 and 4 included ‘following healthcare advice’ and ‘comfort’. ‘Safety and protection of feet’ was reported more frequently at Week 4 compared to Week 0.

### Patient understanding of medical grade footwear

The mean score for patient understanding of MGF before and after pressure analysis at Week 0 is presented for each of the five statements related to MGF use in Table 3 (best possible score 5; worst possible score 1). The overall mean score represents the combined mean for all five statements. The data do not demonstrate large changes in patient understanding with the overall mean score increasing from 4.0 to 4.2 out of 5. With the exception of Statement 3 where the mean score increased from 3.9 pre-assessment to 4.5 post-assessment (*p* = 0.01), the differences between scores for the remaining statements were not statistically significant (*p* > 0.05).

**Table 3:** Patient understanding before and after the intervention at Week 0 (*n* = 15)

**Statement**	**Pre-assessment** ***Mean (SD)***	**Post-assessment** ***Mean (SD)***	***p* value**
1. Because I have diabetes and loss of protective sensation, I may not feel high pressure areas under my feet	4.0 (1.2)	4.4 (0.9)	0.14
2. My footwear and orthotics have been designed to prevent another foot ulcer	4.7 (0.5)	4.7 (0.6)	0.58
3. High pressure areas under my feet may lead to an ulcer if I do not wear my prescribed footwear most (> 80%) of the time	3.9 (1.2)	4.5 (0.8)	0.01^a^
4. Regular shoes may not protect my feet as well as my prescribed footwear	4.1 (1.2)	4.1 (1.3)	1.00
5. It is OK (or safe) to wear slippers or other slip-on house shoes when I am at home as these protect my feet enough	3.1 (1.6)	3.3 (1.5)	0.26
**Overall mean score**	4.0	4.2	-

^a^Indicates statistical significance (*p* < 0.05)

### Satisfaction with medical grade footwear

There was no significant change in the proportion of participants who reported that they were or were not satisfied with medical grade footwear before and after the intervention. Prior to the intervention, 10 participants were satisfied, one was not, three were unsure, and one preferred not to answer. Following the intervention, the number who were satisfied increased to 11, while one remained not satisfied, two unsure and one participant still preferred not to answer. Several themes for satisfaction and dissatisfaction with MGF were identified in the responses to the Pre-assessment Questionnaire B and Post-assessment Questionnaire (see Additional file 3). Similar to the themes surrounding adherence to MGF, ‘comfort’ was a dominant theme for satisfaction with MGF and ‘designed to protect feet and prevent foot complications’ was more likely to be reported following the intervention.

### Experience of in-shoe pressure analysis

Responses to the Week 0–1 Telephone Questionnaire are provided in Table 4, and the themes identified from the questionnaire in Additional file 4. The patient experience of in-shoe pressure analysis was largely positive, with all 15 participants reporting that results were communicated in a way they could understand and attending was worthwhile. When asked why pressure analysis was worthwhile, the primary reasons provided were that it demonstrated pressure points and improved offloading.

**Table 4:** Responses from Week 0–1 Telephone Questionnaire (*n* = 15)

**Question**	**Yes** **n (%)**	**No** **n (%)**
Were the results of your pressure analysis communicated in a way you could understand?	15(100%)	0(0%)
Was attending this appointment worthwhile for you?	15(100%)	0(0%)
Were there any aspects of pressure analysis you found useful or interesting?	13(86.7%)	2(13.3%)
Were there any aspects of pressure analysis you did not enjoy?	0(0%)	15(100%)
Would you be prepared to undergo in-shoe pressure analysis again?	15(100%)	0(0%)

### Adverse events

No adverse events occurred during the in-shoe pressure analysis session at Week 0. Four adverse events occurred over the course of the final study, of which three were attributed to use of medical grade footwear, consisting of a plantar forefoot fissure, dorsal skin trauma and dorsal neuropathic ulcer. The fourth adverse event consisted of haemorrhagic callus at the plantar hallux related to intrinsic foot deformity, not use of MGF. None of the participants were withdrawn from the study or follow-up questionnaires. Three participants were advised to cease use of MGF during the study due to risk of skin trauma and/or ulceration and returned to alternative footwear (*n* = 2) or offloading device (*n* = 1).

## Discussion

### Adherence to medical grade footwear

The primary aim of this study was to determine if patient adherence to MGF improved following in-shoe pressure analysis and modification of MGF. The results of this study indicated that self-reported adherence inside the home increased for 46.7% of participants, decreased for 6.6%, and stayed the same for 46.7%. However, the number of participants who reported ≥ 80% adherence to MGF inside the home was low, increasing from 13.3% at Week 0 to 20.0% at Week 4. Adherence outside the home increased for 33.3% of participants and stayed the same for 66.7%. However, baseline adherence outside the home was high compared to previous research, with 53.3% (*n* = 8) participants reporting ≥ 80% adherence, compared to 26% (*n* = 13) reported by Macfarlane and Jensen [11].

When examining the number of participants who reported ≥ 80% adherence to MGF outside the home, this increased from eight (53.3%) at Week 0 to 12 (80.0%) at Week 4, which indicates a clinically significant improvement in adherence (reaching ≥ 80%) for four participants. In contrast, while adherence inside the home improved for seven participants (46.7%), adherence only increased to a clinically significant level (≥ 80%) for one participant. The finding of low adherence to MGF inside the home compared to outside the home is consistent with the results of previous studies. Macfarlane and Jensen [11] reported that 12% (*n* = 6) of participants wore prescribed MGF > 80% of the time at home compared to 26% (*n* = 13) outside the home, while Waaijman et al. [10] reported mean adherence of 61% at home compared to 87% adherence away from home for 107 participants with previous DFU, and this difference was statistically significant (*p* < 0.01). The challenge of comparing the findings of the current study with previous research is that adherence is not frequently reported separately for indoor and outdoor use, however, this is important given the significant difference in reported adherence between these two conditions [23].

We are not aware of any previous research investigating in-shoe pressure analysis as a tool to improve patient adherence to MGF. Bus et al. [8] and Abbott et al. [24] reported patient adherence following in-shoe pressure analysis, however, the study aims and application of in-shoe pressure analysis were different from the current study. Bus and colleagues sought to blind participants to the intervention by evaluating the results of pressure analysis and modifying footwear out of view, which prevented bias from differences in how groups perceived treatment. The findings of the current study indicate that communicating the results of in-shoe pressure analysis and demonstrating the offloading capacity of MGF may improve adherence. However, a larger study incorporating an objective and accurate measure of adherence is needed to explore this further. For example, Bus et al. [8] utilised an in-shoe temperature monitor combined with a step activity monitor around the ankle. Alternatively, dual accelerometers worn on the body and attached to the shoe can provide an objective measure of adherence [23].

Abbott et al. [24] studied patient adherence to the use of an intelligent insole inserted into MGF, which provided an alert via smartwatch when excessive plantar pressures were detected and prompted participants to modify weight-bearing behaviour. Abbott and colleagues reported that 69% of participants were ‘good compliers’ (defined as a mean of ≥ 4.5 h wear per day). When analysing adherence between the intervention group and control group (which received no alerts), there was no difference in the percentage of ‘good compliers’ between groups (both 69%). However, Bus [25] highlighted the high drop-out rate of 35% during the wearing-in period and 50% in the intervention group during follow-up, which Abbot and colleagues attributed to difficulty adapting to the smartwatch technology, as opposed to the higher frequency of alerts. This notion is supported by the findings of Najafi et al. [26], who conducted a study assessing adherence to the use of the same intelligent insole system and found that adherence increased for those participants who received a high number of alerts per hour compared to those who received a low number of alerts. However, while the current study assessed the impact of a single session of in-shoe pressure analysis measuring mean peak pressure during walking, the insole system assessed by Abbott and colleagues and Najafi et al. [26] consisted of a continuous monitoring system that measured sustained low pressures (around 35 mmHg) during daily activities including sitting, standing and walking. This therefore limits the capacity to compare the findings of these studies to the present study.

While this study demonstrated that adherence outside the home improved to ≥ 80% for four participants at Week 4, it is not known whether the improved adherence would have been sustained at 12 weeks or beyond. Keukenkamp et al. [27] studied the impact of motivational interviewing on footwear adherence in 13 patients with a history of DFU. Participants were randomised to receive either standard education or two 45-min motivational interview sessions. Keukenkamp and colleagues found that adherence outside the home was high at baseline (91% for the intervention group) and remained high at three months (92% intervention group, 93% standard education group). However, adherence inside the home for the intervention group increased from 49 to 84% at week 1, before returning to 40% at three months. This suggests that interventions to modify patient adherence to MGF may need to be repeated to have a sustained impact. This could be explored in a future study where in-shoe pressure analysis and visual demonstration of findings is repeated at monthly intervals, and where adherence is measured at three and six months to determine if repeated sessions provide a sustained impact on adherence.

### Patient understanding of medical grade footwear

The potential for in-shoe pressure analysis to modify patient understanding of the role of high pressure areas and improve adherence was suggested by Najafi et al. [28]. However, Najafi and colleagues were referring specifically to an intelligent insole system providing continuous feedback. The current study sought to investigate the impact of a single session of in-shoe pressure analysis on patient understanding. For the Pre-assessment Questionnaire B and Post-assessment Questionnaire, the mean score for Statement 3 increased from 3.9 to 4.5 and the difference in scores was statistically significant (*p* < 0.05). As Statement 3 referred specifically to using MGF > 80% of the time to avoid ulceration, this indicates that the intervention may have increased participants’ understanding of the need to wear MGF most of the time. However, the scores for the remainder of the statements did not demonstrate a statistically significant difference. Patient understanding at baseline was already at a high level (4 out of 5), which may explain the lack of statistically significant improvement in scores. As participants had already been fitted with MGF and educated on use prior to enrolment in the study, their level of understanding at study entry was already high. Furthermore, it is possible that the questionnaire was not sensitive to detecting change in patient understanding. Questionnaires with more simplified language, and which participants read and complete themselves may prove more sensitive to detecting change. Further studies are required to develop a reliable and valid tool to measure patient understanding of MGF.

The area in which participants had the poorest understanding was use of slippers or other house shoes at home, which is consistent with previous studies [29, 30]. There was minimal change in responses for this statement before and after a single session of pressure analysis, which is reflected in the minimal change in adherence inside the home and the persistence of themes related to non-adherence at home over the four weeks. The themes identified in this study are consistent with the findings of Paton and colleagues [29] who reported that participants viewed the home as a safe and familiar place where there was a low risk of injury. Participants for the current study also expressed a feeling of safety at home where they reported ‘sitting or keeping their feet up’. Further studies exploring the impact of continuous monitoring insole systems—such as the smartwatch employed by Abbott et al. [24]—on patient understanding of the high plantar pressures experienced with inappropriate footwear use at home may be beneficial. However, this would rely upon patient compliance with transferring of insoles between shoes where alternative footwear is worn at home. A barefoot pressure platform, as utilised by Gurney and colleagues [31], could be employed in future studies to illustrate the pressures to which bare feet are subjected and may be useful to compare with in-shoe pressure analysis to demonstrate the attenuation of pressures with MGF. While this may assist in modifying patient behaviour towards MGF use inside the home, the additional cost of a barefoot pressure platform is a limitation.

### Response rate and patient experience

The response rate for the current study was high, with all 26 patients who were informed of the study expressing interest in learning more, of which 22 agreed to participate. The positive patient engagement with in-shoe pressure analysis was reflected in participant responses to the Week 0–1 Telephone Questionnaire, which demonstrated that 100% of participants felt the intervention was worthwhile. The dominant reasons provided by participants for why they found attending worthwhile was the ‘demonstration of pressure points’ (*n* = 4) and ‘improved offloading of pressure points’ (*n* = 3). This suggests that participants understood the purpose of in-shoe pressure analysis, were interested to learn more about the offloading properties of their footwear and valued having their footwear optimised.

### Limitations of the study

While the target of 200kPa used in this study is commonly used to guide footwear and orthotic modification in patients with a history of DFU, Jones et al. [32] noted the limited evidence to support the use of this threshold; consisting of two cohort studies [9, 33] and one RCT [8]. It is also recognised that the findings of this study may not be generalisable to those using pressure measurement systems other than the Pedar, as different systems have different numbers of sensors, measurement range and sampling rate, which limits comparison [32].

Bus et al. [5] successfully offloaded all 35 ROIs targeted for pressure reduction in their study, with successful offloading defined as 25% pressure reduction or MPP < 200 kPa. However, 19 of 35 sites (54%) were successfully offloaded according to the criterion of MPP < 200 kPa. In contrast, Waaijman et al. [6] reported 51–59% of sites were successfully offloaded according to the criterion of 25% reduction or < 200 kPa, and attribute the lower percentage compared to Bus et al. [5] to the larger number of regions targeted for modification. It is acknowledged that the pressure reduction results for the current study were lower with 8 of 24 ROIs (33%) offloaded to < 200 kPa. Possibly contributing to this was the absence of a pedorthist to assist with footwear modification, time restraints preventing further orthotic modifications, and the newly established nature of the clinic. However, the aims of the study were to investigate adherence and understanding of medical grade footwear in response to the intervention as it is used in routine clinical practice, therefore, while the aim was to reduce pressure < 200Kpa this was not deemed essential.

A further limitation of the current study was the subjective measure of self-reported adherence. While Bus et al. [8] and Abbott et al. [24] utilised objective methods of measuring adherence, adherence for the current study was measured subjectively and relied upon participants’ recollection of adherence over the previous four weeks. Therefore, the findings for patient adherence should be interpreted with caution given the risk of bias associated with self-reported adherence to MGF [23, 26]. A further limitation of this study was that plantar pressures were measured in a controlled clinical setting that may not reflect the cumulative forces a patient is subject to over a day [28, 34]. Importantly, other forces such as pressure from the dorsum of the shoe and shear pressure were not able to be captured with the in-shoe pressure technology utilised, however, they may contribute to ulcer formation [35].

Limitations of sample size and sampling method also weaken the findings of this study. The sample size was small and was not calculated to determine statistical significance, therefore, the statistical significance of the results related to patient adherence is not known. The sample size for the pilot and final study combined (*n* = 18) was smaller than the anticipated *n* = 20. Contributing to this was the delay in supply of MGF to patients within SLHD related to the COVID-19 pandemic. Furthermore, all 15 participants included in the final study were male, therefore, the findings of this study reflect an entirely male sample and may not be generalisable to female patients.

The questionnaires used for this study were created for the purpose of this study, therefore, the reliability and validity of these questionnaires is unknown. A pilot study was undertaken to assess participant comprehension and acceptability of the questionnaires. However, as these questionnaires were new, there were no previous studies to directly compare the findings to, limiting the conclusions that could be drawn, particularly regarding patient understanding of MGF. Quantifying changes in patient understanding through analysing differences in Likert score responses may not reflect a true change in understanding, and the clinical significance of the change in scores for patient understanding is unknown. The Week 0 questionnaires were delivered verbally by the investigator who conducted the pressure analysis and orthotic modifications. Despite questionnaires being delivered in a standardised format for all participants, there is the possibility of interviewer bias in how participant responses to short answer questions were noted down and at times summarised.

With regards to the adverse events that occurred during the study, three were attributed to use of MGF, two of which consisted of a dorsal lesion. It is possible that increased use of MGF as a result of participation in the study may have contributed to these events. Changes in physical activity could also have contributed, with participants possibly perceiving that they were more protected in MGF, however, this was not measured in the study and warrants investigation in future studies. A limitation of plantar pressure analysis is that dorsal pressures against the shoe are not able to be measured, which highlights the importance of regular visual inspection of the feet while transitioning into MGF.

### Recommendations for clinical practice

This study confirmed previous research findings that have demonstrated poor adherence to MGF inside the home [10, 11, 27, 29, 30]. In light of the preference of patients to use slippers or simple house shoes at home, the provision of a medical grade slipper in addition to outdoor MGF may increase adherence inside the home, which was also recommended by Paton et al. [29]. However, the inevitable challenge with such footwear is the need to offload areas at risk of DFU, which may necessitate footwear features not compatible with a slipper design [36]. Ultimately, an informed discussion between footwear supplier and patient in which patient goals are addressed and any potential compromise reached may result in the best outcomes for the patient. Clinicians may find it useful to employ in-shoe pressure analysis to demonstrate the difference between a patient’s standard indoor footwear and the recommended MGF, as this may further reinforce the benefit of prescribed footwear.

## Conclusion

In summary, this study has demonstrated that a single session of in-shoe pressure analysis with communication of findings may improve patient adherence to MGF, particularly outside the home. A single session of in-shoe pressure analysis did not significantly impact adherence inside the home. Participants rated the intervention as worthwhile and reported that it increased their understanding, however, the change in scores for patient understanding were small and largely non-significant. Interventions such as continuous monitoring systems that patients can use at home may have a greater impact on patient understanding, and further studies are required to demonstrate the role of this technology in improving patient adherence. Further qualitative studies may provide greater insight into the barriers to MGF use at home, and this greater understanding may assist clinicians in prescribing MGF that is worn more frequently and is therefore more effective in preventing recurrence of DFU. The use of a medical grade slipper or house shoe should be considered given the preference for such footwear indoors reported by participants in this study.

## Supplementary Information

**Additional file 1. **Participant characteristics.

**Additional file 2. **Pressure analysis data.

**Additional file 3. **Themes for satisfaction and dissatisfaction with MGF.

**Additional file 4. **Themes from Week 0-1 Telephone Questionnaire (*n*=15).

## Data Availability

Additional data can be requested from the corresponding author (CM). Email: clare.mcdonogh@health.nsw.gov.au.
