# Supplementary material for: Does in-shoe pressure analysis to assess and modify medical grade footwear improve patient adherence and understanding? A mixed methods study
Source: J Foot Ankle Res. 2022 Dec 24;15:94. doi: 10.1186/s13047-022-00600-0 (PMC9789308; doi:10.1186/s13047-022-00600-0)
Supplement: Supplementary file 1 — Additional file 1. Participant characteristics. [file 13047_2022_600_MOESM1_ESM.docx]

**Additional file 1 (DOCX 17kb)**

**Participant characteristics**

| **Characteristic** | **Participant Data** |
| --- | --- |
| Mean age in years (range) | 62.5 (41-86) |
| Sex *n* (%)  Male | 15 (100) |
| Type of diabetes *n* (%)  T1  T2 | 1 (7)  14 (93) |
| Diabetes control *n* (%)  Diet only  Tablets  Insulin  Other injectables  Tablets and insulin/other injectables | 0 (0)  7 (47)  2 (13)  0 (0)  6 (40) |
| Mean duration of diabetes in years (range) | 16.5 (1-39) |
| Average HbA1c% mean (range)  *n*=10 | 7.5 (5.3 - 11.5) |
| Diabetes complications *n* (%)  Retinopathy  Nephropathy  Autonomic neuropathy | 4 (27)  5 (33)  1 (7) |
| Other complications *n* (%)  Hypertension  Dyslipidaemia  Cerebrovascular disease  Ischaemic heart disease  Osteoarthritis  None | 15 (100)  10 (67)  1 (7)  5 (33)  4 (27)  1 (7) |
| Requires dialysis *n* (%)  Yes  No | 1 (7)  14 (93) |
| Smoking status *n* (%)  Current  Previous  Never | 0 (0)  8 (53)  7 (47) |
| Median age of MGF in months (range) | 2.5 (0.5-12) |
| Type of MGF *n* (%)  Fully customised  Modified MGF  *Modifications:*  Rigid forefoot rocker  Leg length discrepancy raise  Prefabricated MGF | 3 (20)  10 (67)  9 (60)  1 (7)  2 (13) |
| First pair of MGF *n* (%)  Yes  No  Unsure | 8 (53)  7 (47)  0 (0) |
| History of in-shoe pressure analysis *n* (%)  Yes  No  Unsure | 0 (0)  15 (100)  0 (0) |
| Location of previous ulcer right foot *n* (%)  Hallux  Lesser digit(s)  Forefoot  Midfoot  Hindfoot | 9 (60)  1 (7)  8 (53)  1 (7)  0 (0) |
| Location of previous ulcer left foot *n* (%)  Hallux  Lesser digit(s)  Forefoot  Midfoot  Hindfoot | 3 (20)  2 (13)  7 (47)  1 (7)  0 (0) |
| Median time since most recent plantar ulcer healed in months (range)  *n=*12 (not known: *n=*1; site amputated: *n=*2) | 9.4 (1 - 180) |
| History of amputation *n* (%)  Yes  No | 5 (33)  10 (67) |
| Type of amputation *n* (%)  Hallux  Lesser digit (single)  Lesser digits (multiple) | 2 (13)  1 (7)  2 (13) |
| Peripheral arterial disease *n* (%)  Yes  No | 2 (13)  13 (87) |
| History of falls in last 12 months *n* (%)  Yes  No | 1 (7)  14 (93) |
| Foot deformity (right and/or left foot)* *n* (%)  *Where participants had the same deformity for both feet, it is reported once.  Hallux abducto valgus  Hallux limitus/rigidus  Claw/hammer toes  Pes cavus  Pes planus  Charcot deformity  Prominent metatarsal heads | 1 (7)  7 (47)  9 (60)  3 (20)  4 (27)  2 (13)  7 (47) |
