# Supplementary material for: Does in-shoe pressure analysis to assess and modify medical grade footwear improve patient adherence and understanding? A mixed methods study
Source: J Foot Ankle Res. 2022 Dec 24;15:94. doi: 10.1186/s13047-022-00600-0 (PMC9789308; doi:10.1186/s13047-022-00600-0)
Supplement: Supplementary file 2 — Additional file 2. Pressure analysis data. [file 13047_2022_600_MOESM2_ESM.docx]

**Additional file 2 (DOCX 17kb)**

**Pressure analysis data**

| **Pressure reduction at all ROI >200 kPa and modifications performed** | | | | | | |
| --- | --- | --- | --- | --- | --- | --- |
| **Participant number** | **Location of ROI** | **MPP at ROI before modification** | **MPP at ROI after modification** | **Reduction in MPP (%)** | **Number of rounds of modifications** | **Type of modification** |
| 1 | Plantar right hallux (proximal to previous ulcer site) | 215 | 123 | 42.8% | 1 | Wedge removed and replaced with 2mm high density EVA layer to forefoot with cut-out to ulcer site. |
| 2 | 1. Central plantar right hallux*  2. Distal plantar right hallux. | 1. 258  2. 578 | 1. 188  2. 575 | 1. 27.1%  2. 0.5% | 3  Site 2 not targeted for further modification due to concern pressure would increase at site 1 (previous ulcer site). | 2mm high density EVA layer with cut-out to right hallux.  Forefoot medial wedge right foot. |
| 3 | Right 3^rd^ metatarsal head | 280 | 228 | 18.6% | 1 | Deflection created in right orthotic under 3^rd^ MTH filled with slow-release poron. |
| 4 | Nil regions > 200kPa | N/A | N/A | N/A | N/A | N/A |
| 5 | 1. Plantar right 1^st^ MTH*  2. Plantar left 1^st^ MTH | 1. 625  2. 365 | N/A | N/A | 0  Advised to cease use of MGF as footwear required modification by pedorthist. | N/A |
| 6 | Left 1^st^ MTH* | 303 | 268 | 11.6% | 2 | 6mm slow-release poron added to left 1^st^ MTH. |
| 7 | 1. Plantar right 1^st^ MTH*  2. Plantar right 3^rd^ MTH  3. Plantar left 4^th^ MTH | 1. 203  2. 248  3. 253 | 1. 145  2. 185  3. 170 | 1. 28.6%  2. 25.4%  3. 32.8% | 3 | 3mm slow release poron added under both forefeet.  Poron metatarsal domes bilaterally.  2mm low density EVA top cover |
| 8 | 1. Right 1^st^ MTH*  2. Right hallux*  3. Left 1^st^ MTH | 1. 270  2. 255  3. 225 | 1. 293  2. 238  3. 213 | 1. -8.5% Increased  2. 6.7%  3. 5.3% | 3 | 1^st^ MTH deflection filled with slow-release poron bilaterally  Plantar right hallux deflection filled with slow-release poron |
| 9 | 1. Plantar left hallux  2. Plantar right hallux | 1. 263  2. 210 | 1. 263  2. 210 | N/A | 0  Site of previous ulcer (plantar left 1^st^ MPJ) <200kPa at baseline. Concern that altering orthotic to reduce pressure at plantar left hallux may alter pressure at previous ulcer site. | N/A |
| 10 | Plantar right 3^rd^ MTH* | 288 | 208 | 27.8% | 3 | Metatarsal dome added and 2mm low-density EVA top cover |
| 11 | Plantar right 1^st^ MTH | 265 | 205 | 22.6% | 3 | Material ground down from front of right orthotic. Slow release poron added to Deflection plantar right 1^st^ MTH. PPT metatarsal bar added to right orthotic. |
| 12 | Nil regions >200kPa | N/A | N/A | N/A | N/A | N/A |
| 13 | 1. Plantar right 1^st^ MTH  2. Plantar left 1^st^ MTH | 1. 230  2. 225 | 1. 203  2. 193 | 1. 11.7%  2. 14.2% | 1 | 3mm bilateral full-length EVA raise. Bilateral metatarsal domes. Bilateral 2mm low density EVA top cover |
| 14 | 1. Plantar right hallux  2. Plantar left 3^rd^ MTH | 1. 280  2. 233 | 1. 188  2. 198 | 1. 32.9%  2. 15.0% | 2 | Full-length high density EVA layer with cut-out plantar right hallux.  Bilateral metatarsal domes.  Bilateral 2mm low-density EVA top cover. |
| 15 | 1. Plantar right hallux*  2. Plantar left hallux  3. Plantar right 1^st^ MPJ | 1. 248  2. 243  3. 250 | 1. 215  2. 208  3. 265 | 1. 13.3%  2. 14.4%  3. -6.0% Increased | 2 | Full length 2mm high-density EVA layer with cut-out to hallux bilaterally. |

*previous ulcer site
