# Supplementary material for: Does in-shoe pressure analysis to assess and modify medical grade footwear improve patient adherence and understanding? A mixed methods study
Source: J Foot Ankle Res. 2022 Dec 24;15:94. doi: 10.1186/s13047-022-00600-0 (PMC9789308; doi:10.1186/s13047-022-00600-0)
Supplement: Supplementary file 3 — Additional file 3. Themes for satisfaction and dissatisfaction with MGF. [file 13047_2022_600_MOESM3_ESM.docx]

**Additional file 3 (DOCX 14kb)**

**Themes for satisfaction and dissatisfaction with MGF**

| **Themes for satisfaction with MGF** | **Number of times theme identified** | |
| --- | --- | --- |
|  | **Before intervention** | **After intervention** |
| Designed to protect feet and prevent foot complications e.g. ulcer  Helps to prevent corns and calluses  Comfort  Prevents pain  Confidence in walking/maintaining mobility  Ease of application  Personalisation/customisation  Appropriate fit  Appearance/design  Accustomed to use  Appreciative of resources  Perceived benefit/value  **Total** | 2  1  4  1  2  1  1  1  0  0  0  3  **16** | 4  0  4  1  3  1  0  1  1  1  2  1  **19** |
| **Themes for dissatisfaction with MGF** |  |  |
| Stability concerns  Difficulty applying and removing  Difficulty driving  Heaviness  Deterioration of materials  Issues with fit  Difficulty adjusting to MGF  Appearance  Discomfort  Lack of perceived benefit  **Total** | 2  1  1  1  3  2  1  2  1  1  **15** | 1  3  1  0  1  2  2  0  0  0  **10** |
