# Supplementary material for: Does in-shoe pressure analysis to assess and modify medical grade footwear improve patient adherence and understanding? A mixed methods study
Source: J Foot Ankle Res. 2022 Dec 24;15:94. doi: 10.1186/s13047-022-00600-0 (PMC9789308; doi:10.1186/s13047-022-00600-0)
Supplement: Supplementary file 4 — Additional file 4. Themes from Week 0-1 Telephone Questionnaire (n=15). [file 13047_2022_600_MOESM4_ESM.docx]

**Additional file 4 (DOCX 13kb)**

**Themes from** **Week 0-1 Telephone Questionnaire (*n*=15)**

| **Themes for pressure analysis being worthwhile** | **Number of times theme identified** |
| --- | --- |
| Demonstrated pressure points | 4 |
| Ability to adjust MGF on the spot | 1 |
| Improved offloading of pressure points | 3 |
| Demonstrated effectiveness of MGF | 1 |
| Maintaining mobility | 1 |
| Generating knowledge to guide future footwear | 1 |
| **Themes for what participants found useful or interesting** |  |
| Demonstrated pressure points | 3 |
| Demonstrated pathology related to pressure points | 3 |
| Ability to adjust MGF on the spot | 3 |
| Improved offloading of pressure points | 2 |
| Demonstrated effectiveness of MGF | 2 |
| Interesting | 2 |
| Seeing pressure images on the screen | 2 |
| Improved comfort of MGF | 2 |
